# Stepwise dehydration of Cd-exchanged levyne: thermal stability and structural modifications

**DOI:** 10.1007/s00269-021-01146-6

**Published:** 2021-05-12

**Authors:** Georgia Cametti

**Affiliations:** Institute of Geological Sciences, Baltzerstrasse 1+3, 3012 Bern, Switzerland

**Keywords:** Levyne, Cd-zeolite, X-ray diffraction, Thermal stability

## Abstract

Zeolites show remarkable properties that can be tuned through cation exchange of their original extraframework content. In this respect, the response of the modified zeolite to the heating stimuli, in terms of structural modifications and thermal stability, can drastically change and is, therefore, an important factor to consider. In this study, the dehydration mechanism of a natural levyne previously exchanged with Cd^2+^ has been monitored in situ by single crystal X-ray diffraction. The initial dehydration trend between 50 and 175 °C is similar to that observed for the pristine material, levyne-Ca. The water loss is accompanied by extraframework cation migration within the zeolitic cavities and the unit-cell volume slightly contracts from 3503.8(1) to 3467.8(6) Å^3^. From 200 to 250 °C, a pronounced drop of the unit-cell volume (− 7%) is observed. The dehydrated structure at 250 °C corresponds to levyne B topology of natural levyne, characterized by the statistical rupture of the T–O–T bonds of the double six-ring membered cage. However, in contrast to levyne-Ca, the fraction of broken connections reached 50% instead of 37%, and no additional structural modifications were detected up to 350 °C. At 400 °C, diffraction data pointed to the onset of the structural collapse. At this temperature, the measured unit-cell volume was 8% smaller compared to that of the RT structure. The corresponding contracted structure did not rehydrate after exposure to humid conditions for 21 days.

**Supplementary Information:**

The online version contains supplementary material available at 10.1007/s00269-021-01146-6.

## Introduction

The mineral levyne belongs to the group of zeolites. At room temperature (RT), levyne is rhombohedral, space group *R*-3* m*, a_hex_ = 13.35 *c*_hex_ = 22.90 Å, V = 3534 Å^3^ (Gottardi and Galli 1985). The structure consists of interconnected tetrahedra TO_4_ (Ti = Si^+4^, Al^3+^), which form single and double-membered rings stacked along the c-axis according to the AABCBBCAA sequence. The **LEV** framework type (Baerlocher et al. 2007) is built up by the so-called *lev* cavity [4^9^6^5^8^3^], which alternates with double six-rings (D6R) polyhedra [4^6^6^2^] along [001]. Two 8-memebered ring channels run perpendicular to this direction. The dominant extraframework (EF) cations are Ca and Na (Coombs et al. 1997), distributed along the threefold axis at different crystallographic positions that can vary depending on the sample composition (Sacerdoti 1996; Gatta et al. 2005; Arletti et al. 2013; Ballirano and Cametti 2013).

The response of zeolites to external stimuli such as temperature, pressure and water vapour pressure depends on many factors and can significantly change according to the applied experimental conditions (Cruciani et al. 2006). In the past, the crystal structure of levyne-Ca was investigated at high pressure and high temperature. Gatta et al. (2005) and Gatta and Well (2006) reported an anomalous elastic behaviour of levyne-Ca, characterized by a change in the compression mechanism at *P* > 1GPa. At high temperature, natural levyne experiences different transformations as a function of dehydration. These involve the contraction of the unit-cell volume, the statistical rupture of tetrahedral bonds T–O–T constituting the framework and the migration of Ca atoms within the zeolitic cavities (Arletti et al. 2013). Such transformations lead to the formation of two new structural topologies, levyne B (Arletti et al. 2013) and levyne B’ (Cametti 2018) with different stacking sequence compared to the RT structure. The extent of the contraction of the unit-cell volume and the type of structural topology, which forms upon dehydration, depend on the experimental set-up and on the metal cations, which occupy the zeolitic cavities. For instance, levyne B’ topology was observed only during in situ single crystal X-ray diffraction (SC-XRD) experiments at 275 °C, when the rupture of an additional T–O–T link of the framework began. In contrast, this transformation was not reported in the synchrotron X-ray powder diffraction (SR-XRPD) study, where levyne B is maintained up to 700 °C (Arletti 2013). Moreover, recent studies on a fully Ag-exchanged natural levyne (Ag-LEV) (Cametti et al. 2020) indicated that with only Ag^+^ as EF cation, the mineral undergoes a different dehydration path compared to levyne-Ca. Upon heating, the contraction of the unit-cell volume is less pronounced (4% vs 5% for Ca- and Ag-LEV, respectively) and the levyne B’ topology, under the same experimental conditions used for levyne-Ca, does not form.

In the frame of a research aimed at investigating the role of the EF cations on the thermal stability of natural zeolites, we recently reported on the crystal structure of levyne after Cd-exchange in aqueous solution (Cametti et al. 2021). The RT structure of Cd-exchanged levyne (Cd-LEV), with chemical composition Cd_2.6_Ca_0.6_Al_6.6_Si_11.4_O_36_·18.0H_2_O, can still be described in *R*-3* m* space group and the distribution of the EF cations in the pores resembles that typical of levyne-Ca.

In the present study, the thermal stability of this Cd-exchanged levyne is investigated by means of single crystal X-ray diffraction (SC-XRD) experiments. The dehydration was followed in situ in order to track the structural modifications occurring upon heating. The results are then compared with those reported for the natural counterpart, levyne-Ca (Cametti 2018) and for the Ag-exchanged form, Ag-LEV (Cametti et al. 2020).

## Experimental methods

The sample used in this study was the same described in Cametti et al. (2021) with average chemical composition Cd_2.6_Ca_0.6_Al_6.6_Si_11.4_O_36_·18.0H_2_O. A single crystal of Cd-LEV was glued on the top of a glass fibre and mounted on a goniometer head. X-ray diffraction experiments were carried out using a Bruker Apex II diffractometer equipped with a CCD detector, a MoKα radiation (*λ* = 0.71073 Å) and an in house-built temperature regulated N_2_ blower. Diffraction data were collected from RT to 400 °C in steps of 25 °C. The crystal was equilibrated for at least 30 min before starting each data collection. Such experimental conditions can be regarded as dry and “close to equilibrium” and were the same applied in the high temperature (HT) study of the corresponding pristine material, levyne-Ca (Cametti 2018) and of the Ag-exchanged levyne (Cametti et al. 2020).

The Apex3 software package (Bruker AXS 2019) was used for the data reduction and absorption correction. Structure solution was performed by direct methods using the software ShelxT (Sheldrick 2008). Structural refinements of each data set were carried out by Shelxl 2014 (Sheldrick 2015) using neutral atomic scattering factors. The extraframework species were located for each data set by difference-Fourier maps. Obverse–reverse twinning [− 100 0–10 001] was observed in all data sets. From RT to 100 °C, the data were refined in space group *R*-3*m* (Merlino et al. 1975; Cametti, 2018). From 100 °C on, the analysis of the systematic extinctions and *R*_sym_ values as determined by Xprep (Bruker AXS 2019) suggested space group *R*-3. However, due to the small differences between the R factors indicator of *R*-3* m* and *R*-3, test refinements were performed in both space groups. Finally, based on the *R*_int_, R1 and weighting scheme values of the structural refinements, *R*-3*m* was maintained from 100 to 125 °C. Data sets collected at 150 and 175 °C were refined in space group *R*-3. Crystallographic sites corresponding to symmetry-equivalent positions in *R*-3*m* were labelled by double digit (e.g. T1 and T11 in *R*-3 correspond to T1 in *R*-3*m*). Between 200 and 250 °C the quality of the diffraction data, in terms of broadening and smearing of the reflections, significantly decreased leading to *R*_int_ values > 10%. Hence, in this temperature range only the unit-cell parameters were extracted. At 275 °C, although the maximum resolution drastically dropped (maximum 2theta = 46.24°) it was possible to solve and refine the structure in *R*-3 space group. In this structure, the new sites T1B, T11B, OB1 and OB2 appeared. These sites were interpreted due to statistical rupture of the T1–O2–T11 and T1–O3–T11 connections (see “Results” section). T1B and T11B were refined with Si scattering factors whereas OB1 and OB2 with O scattering factors. The occupancy of T1, T11, T1B, T11B, O2, O3, OB1 and OB2 was at first refined without constraints. After a first cycle of refinement, the occupancies of T1 and T11 converged to similar values and they were then constrained to be equal. Similarly, T1B and T11B occupancies approached (1-Occ.T1). A similar strategy was used to refine the population of the oxygen atoms O2, O3, OB1 and OB2 involved in the T–O–T breaking process. Finally, the following constraints were used: (Occ.T1) = (Occ.T11) = (1−Occ.T1B) = (1−Occ.T11B) = (Occ.OB1) = (1−Occ.OB2) = (Occ.O3) = (1−Occ.O2).

At 400 °C, only the cell parameters were determined. The rehydration capacity of the dehydrated crystal used in SC-XRD measurements was tested by exposing it to humid conditions for 21 days at RT. After that, a new set of X-ray data was measured with the same diffractometer used for the HT experiments. Crystal data and refinement details of the structures at RT, 50, 100, 150, 175 and 275 °C are summarized in Table 1. All structural drawings have been produced by the VESTA software (Momma and Izumi 2011). Crystallographic information files are provided as supporting information.

**Table 1:** Crystal data and refinement parameters of Cd-LEV at RT, 50, 100, 150, 175 and 275° C

Crystal data	Cd-LEV RT	Cd-LEV 50	Cd-LEV 100	Cd-LEV 150	Cd-LEV 175	Cd-LEV 275
*a* (Å)	13.4199(19)	13.3930(7)	13.2593(6)	13.1451(6)	13.1177(9)	12.6260(11)
*c* (Å)	22.465(5)	22.3519(15)	22.7333(14)	23.1673(13)	23.271(2)	23.513(2)
*V* (Å^3^)	3503.8(12)	3472.2(4)	3461.3(4)	3466.8(4)	3467.8(6)	3246.1(6)
*Z*	3	3	3	3	3	3
Space group	*R-*3* m*	*R-*3* m*	*R-*3* m*	*R-*3	*R-*3	*R-*3
Refined chemical formula	Cd_1.81_Ca_0.96_(Si, Al)_18_O_36_·21 H_2_O	Cd_2.03_Ca_0.92_(Si, Al)_18_O_36_·18 H_2_O	Cd_1.70_Ca_1.03_(Si, Al)_18_O_36_·11 H_2_O	Cd_1.93_Ca_0.93_(Si, Al)_18_O_36_·4.7H_2_O	Cd_2.17_Ca_0.88_(Si, Al)_18_O_36_·0.3H_2_O	Cd_2.10_Ca_1.24_(Si, Al)_18_O_36_
Crystal size (mm)	0.10 × 0.12 × 0.13	0.10 × 0.12 × 0.13	0.10 × 0.12 × 0.13	0.10 × 0.12 × 0.13	0.10 × 0.12 × 0.13	0.10 × 0.12 × 0.13
Intensity measurement						
Diffractometer	BRUKER APEX II SMART	BRUKER APEX II SMART	BRUKER APEX II SMART	BRUKER APEX II SMART	BRUKER APEX II SMART	BRUKER APEX II SMART
X-ray radiation	MoKα λ = 0.71073 Å	MoKα λ = 0.71073 Å	MoKα λ = 0.71073 Å	MoKα λ = 0.71073 Å	MoKα λ = 0.71073 Å	MoKα λ = 0.71073 Å
X-ray power	50 kV, 30 mA	50 kV, 30 mA	50 kV, 30 mA	50 kV, 30 mA	50 kV, 30 mA	50 kV, 30 mA
Monochromator	Graphite	Graphite	Graphite	Graphite	Graphite	Graphite
Temperature (°C)	25	50	100	150	175	275
Exposure time (s)	10	10	10	10	10	10
Max. 2*θ* (°)	54.53	55.58	52.52	51.58	49.61	47.96
Index ranges	− 15 ≤ *h* ≤ 14	− 15 ≤ *h* ≤ 14	− 15 ≤ *h* ≤ 13	− 15 ≤ *h* ≤ 13	− 15 ≤ *h* ≤ 13	− 14 ≤ *h* ≤ 14
	− 17 ≤ *k* ≤ 17	− 17 ≤ *k* ≤ 17	− 16 ≤ *k* ≤ 16	− 15 ≤ *k* ≤ 15	− 15 ≤ *k* ≤ 15	− 14 ≤ *k* ≤ 14
	− 28 ≤ *l* ≤ 26	− 26 ≤ *l* ≤ 29	− 28 ≤ *l* ≤ 26	− 28 ≤ *l* ≤ 26	− 27 ≤ *l* ≤ 25	− 26 ≤ *l* ≤ 24
No. of measured reflections	10,576	10,646	10,118	9839	9259	8412
No. of unique reflections	989	1036	897	1484	1332	1135
No. of observed reflections *I* > 2*σ* (*I*)	897	912	774	1169	1042	610
Structure refinement						
No. of parameters used in the refinement	79	79	94	135	114	90
*R*(int)	0.0625	0.0530	0.0536	0.0663	0.0614	0.0884
*R*(*σ*)	0.0304	0.0281	0.0261	0.0445	0.0431	0.0614
GooF	1.176	1.176	1.134	1.139	1.124	1.476
R1, *I* > 2*σ* (*I*)	0.0609	0.0556	0.0712	0.0798	0.0861	0.1568
R1, all data	0.0662	0.0622	0.0799	0.0973	0.1040	0.2108
wR2 (on *F*^2^)	0.1853	0.1621	0.1981	0.2229	0.2444	0.4367
Δ*ρ*_max_ (eÅ^−3^) close to	1.26 C5	2.23 C5	0.90 W4	0.82 C6	0.98 C8	0.98 C6
Δ*ρ*_min_ (− eÅ^−3^) close to	− 0.75 C4	− 1.26 C5	− 1.48 C1A	− 1.06 W3	− 0.93 CW2A	− 1.30 C3
BASF	0.074(6)	0.057(5)	0.074(8)	0.108(8)	0.138(9)	–

## Results and discussion

The crystal structure of Cd-LEV at RT (Table S1) is in agreement with that determined in our previous study (Cametti et al. 2021). Minor differences are found in the distribution of the EF cations. C1 is split over two positions, C1 (Occ. = 0.32(8)) and C1A (Occ. = 0.48(8)), with total occupancy equal to that determined for C1 in Cametti et al. 2021. Ca atoms are found at C3 site and, in addition, at C4 site (not present in Cametti et al. 2021). This tiny rearrangement of the Cd and Ca atoms also lead to a shifted position of the H_2_O at the W3 site, whereas no significant differences are found for W1, W2 and W4. Their atomic coordinates are in agreement with those previously reported for Cd-LEV (Cametti et al. 2021).

### Unit-cell parameter variation as a function of temperature

**Fig. 1:**
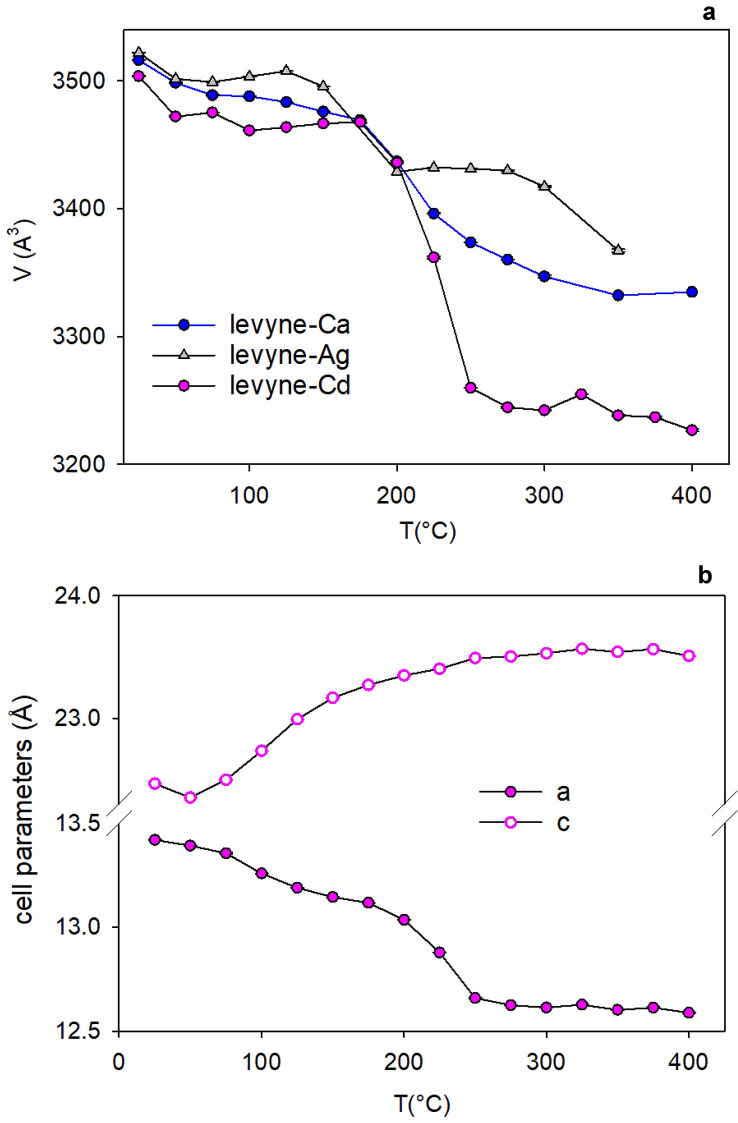
Unit-cell volume (**a**) and cell parameters (**b**) trend of Cd-exchanged levyne as a function of increasing temperature. Corresponding values of natural levyne (levyne-Ca) (Cametti 2018) and Ag-exchanged levyne (Cametti et al. 2020) are also displayed (**a**) for comparison

The change of the unit-cell parameters as a function of temperature is reported in Fig. 1. Corresponding values of the unit-cell volume trend of levyne-Ca (Cametti 2018) and Ag-LEV (Cametti et al. 2020) are also shown for comparison (Fig. 1a). Upon heating, the simultaneous expansion and contraction along the c- and a-axis (Fig. 1b), respectively, causes an overall decrease of the unit-cell volume. At first, a tiny contraction occurs from 25 to 125 °C, when the volume decreases by approximately 1% if compared to that measured at RT. A more pronounced change is observed form 175 to 250 °C, when the reduction amounts to 7%. Such value is significantly higher compared to that of both levyne-Ca and Ag-LEV at corresponding temperatures. As a reminder, the data reported in this study were collected under the same experimental conditions used for the other cationic forms. At 400 °C, the unit-cell volume further decreases, reaching a total contraction of 8% with respect to the RT structure.

### Structural modifications upon heating

**Fig. 2:**
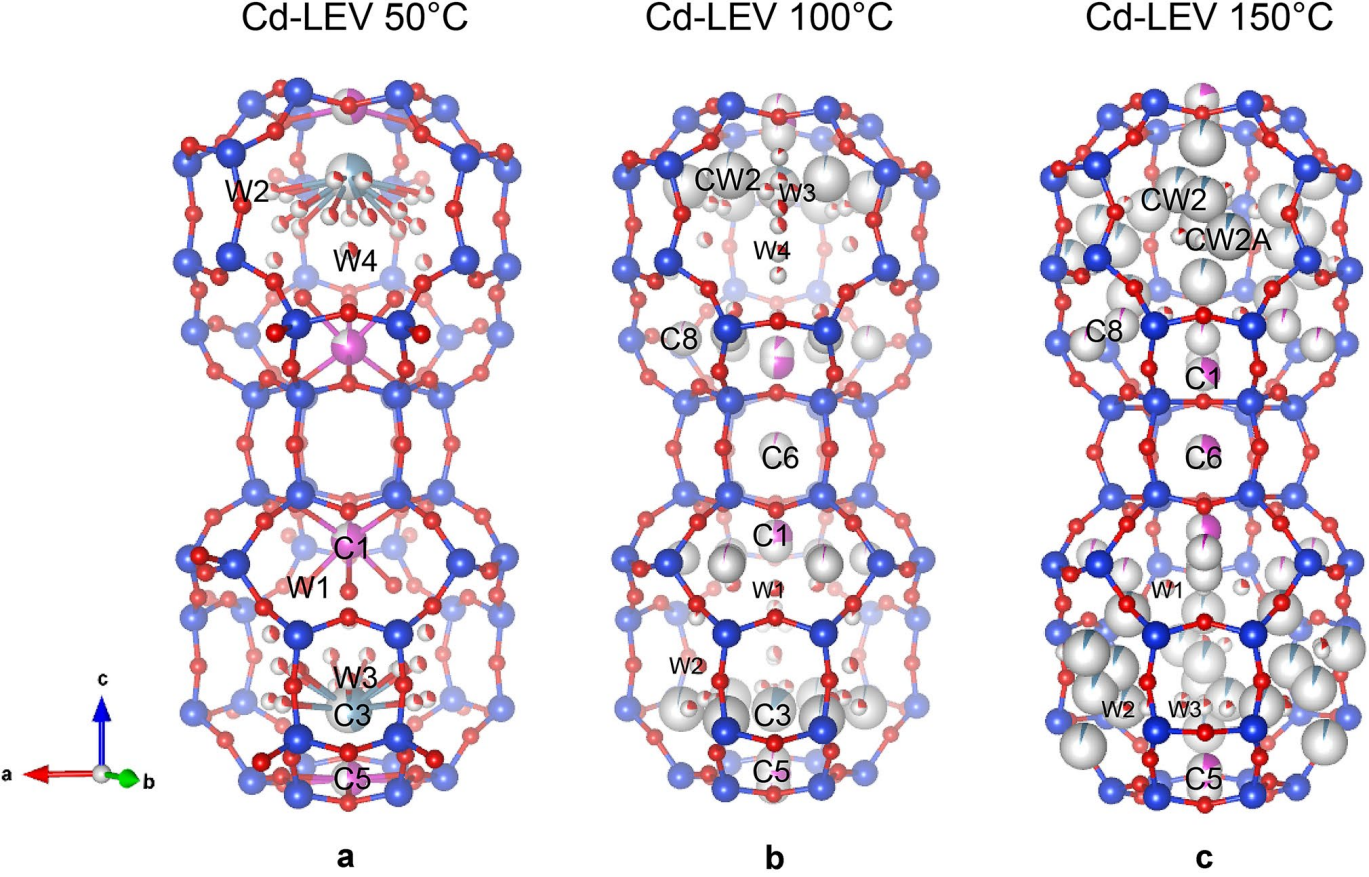
Crystal structures of Cd-exchanged levyne at 50, 100 and 150 °C. Si and O atoms are reported as blue and red spheres, respectively. Cd atoms are displayed in pink and Ca in light blue. Partial colour of the spheres corresponds to the refined partial-occupancy of the crystallographic sites

The dehydration of Cd-exchanged levyne starts at 50 °C, when part of the H_2_O at W2, W3 and W4 sites is released. In contrast, the W1 site remains fully occupied (Table 2). The water loss is accompanied by a change in the Ca distribution and Ca completely migrates to the C3 site (Occ. = 0.46(3)), emptying C4. The H_2_O at W2, W3 and W4 also readjust their positions, which result in a more disordered configuration (Fig. 2a).

**Table 2:** Atom coordinates, occupancy and atomic displacement parameters of Cd-LEV at 50 °C

Site	Scattering factor	*x*	*y*	*z*	Occ	*U*^eq^ (*Å*^2^)
T1	Si	0.43827(11)	0.10482(11)	0.40503(6)	1	0.0156(4)
T2	Si	0.23721(13)	0	0.5	1	0.0142(5)
O1	O	0.3168(3)	0.0251(4)	0.44051(18)	1	0.0321(10)
O2	O	0.4937(5)	0.2469(3)	0.4206(2)	1	0.0273(12)
O3	O	0.5367(2)	0.0735(5)	0.4282(2)	1	0.0260(12)
O4	O	0.4122(5)	0.0789(5)	0.3333	1	0.0295(13)
O5	O	0.1085(3)	− 0.1085(3)	0.4845(3)	1	0.0321(13)
Extraframework						
C1	Cd	0.6667	0.3333	0.47716(5)	0.785(7)	0.0260(5)
C3	Ca	0	0	0.3962(12)	0.46(3)	0.165(13)^a^
C5	Cd	0	0	0.5	0.460(8)	0.0217(10)^a^
W1	O	0.5922(3)	0.1843(7)	0.5470(4)	1	0.070(3)
W2	O	0.1507(13)	− 0.0829(13)	0.3767(7)	0.300(19)	0.043(6)^a^
W2A	O	0.144(4)	− 0.052(4)	0.327(2)	0.37(2)	0.2^b^
W3	O	0.161(5)	0.080(3)	0.343(3)	0.29(4)	0.13(3)^a^
W4	O	0.125(3)	− 0.125(3)	0.276(3)	0.39(4)	0.2^b^

^a^U isotropic

^b^Fixed

The dehydration proceeds with the gradual depletion of the W1, W2, W3 and W4 sites and at 100 °C, the content of structural H_2_O decreases to 11 H_2_O pfu (Table 3). This decrease is accompanied by an increase of the disorder of the extra framework species. Ca and Cd start migrating toward the wall of the *lev* cavity at the CW2 and C8 sites, respectively (Fig. 2b). However, the high structural disorder makes complex the unequivocal assignment of Ca and H_2_O, which spread over low occupied sites close to each other (Table 3). From this temperature on, electron density was detected at the centre of the double six-membered ring cages refined with Cd atomic scattering factor. The position of this site corresponds to that of C6 and Ag1 in the high-temperature structures of levyne-Ca (Cametti 2018) and Ag-LEV (Cametti et al. 2020), respectively. Upon heating, the occupancy at C6 gradually increases, indicating that, similar to levyne-Ca and Ag-LEV, part of the EF cations approaches the D6R due to dehydration.

**Table 3:** Atom coordinates, occupancy and atomic displacement parameters of Cd-LEV at 100 °C

Site	Scattering factor	*x*	*y*	*z*	Occ	*U*^eq^ (*Å*^2^)
T1	Si	0.43342(15)	0.10003(16)	0.40245(7)	1	0.0230(6)
T2	Si	0.23966(17)	0	0.5	1	0.0194(6)
O1	O	0.3175(5)	0.0178(5)	0.4416(2)	1	0.0500(16)
O2	O	0.4866(8)	0.2433(4)	0.4168(4)	1	0.049(2)
O3	O	0.5362(4)	0.0723(8)	0.4240(3)	1	0.046(2)
O4	O	0.4015(7)	0.0681(7)	0.3333	1	0.046(2)
O5	O	0.1104(4)	− 0.1104(4)	0.4884(4)	1	0.053(2)
Extraframework^a^						
C1	Cd	0.6667	0.3333	0.4671(6)	0.53(5)	0.0349(14)
C1A	Cd	0.6667	0.3333	0.481(3)	0.11(5)	0.028(8)
C3	Ca	0	0	0.400(2)	0.13(2)	0.074(18)
C5	Cd	0	0	0.5	0.29(3)	0.023(3)
C5A	Cd	0	0	0.481(3)	0.049(16)	0.009(12)
C6	Cd	0.6667	0.3333	0.3333	0.039(7)	0.005(11)
C8	Cd	0.428(3)	0.093(3)	0.5001(17)	0.013(3)	0.030(14)
CW2	Ca	0.239(5)	0.000(5)	0.398(2)	0.026(7)	0.029(19)
W1	O	0.5927(8)	0.1854(15)	0.5422(7)	0.64(3)	0.069(7)
W2	O	0.129(3)	− 0.129(3)	0.326(3)	0.40(3)	0.2^b^
W2A	O	0.144(2)	− 0.144(2)	0.3887(19)	0.24(4)	0.064(19)
W4	O	0.201(3)	− 0.201(3)	0.414(3)	0.12(3)	0.04(2)
W3	O	0.076(2)	− 0.1399(18)	0.3784(9)	0.24(2)	0.040(9)

^a^U isotropic

^b^fixed

At 150 °C, a significant relocation of the EF cations takes place. Ca at C3 completely moves to the middle of the eight-membered ring windows of the *lev* cavity, at the CW2, CW2A and CW2B sites (Table 4, Fig. 2c). In addition, the migration of Cd inside the D6R cage continue and the occupancy of C6 reaches 30%. Residual H_2_O content (approximately 5 H_2_O pfu) is retained at W1, W2, W2B and W3 sites. At 175 °C, the increasing structural disorder hampers a clear determination of the EF-cations/H_2_O distribution. The refined content of structural H_2_O amounts to 0.3 H_2_O pfu that is found exclusively at W2 site. However, it cannot be excluded that some H_2_O might be present also at the CW2 and CW2A sites (Table 5), as suggested by the slightly higher value of refined Ca atoms (i.e. 0.9 vs 0.6 apfu based on the chemical composition of this sample).

**Table 4:** Atom coordinates, occupancy and atomic displacement parameters of Cd-LEV at 150 °C

Site	Scattering factor	*x*	*y*	*z*	Occ	*U*^eq^ (*Å*^2^)
T1	Si	0.66709(18)	0.09596(19)	0.89947(9)	1	0.0340(6)
T11	Si	0.57114(19)	0.90418(19)	0.10057(9)	1	0.0340(6)
T2	Si	0.75980(16)	0.00016(17)	0.99997(9)	1	0.0294(6)
O1	O	0.7037(7)	0.0238(7)	0.9434(3)	1	0.067(2)
O11	O	0.6802(6)	0.9766(7)	0.0567(3)	1	0.066(2)
O2	O	0.7595(6)	0.5190(7)	0.9021(4)	1	0.083(3)
O3	O	0.5379(5)	0.0753(6)	0.9175(3)	1	0.0538(17)
O4	O	0.6669(5)	0.0512(6)	0.8334(2)	1	0.0520(15)
O5	O	0.5568(6)	0.1138(8)	0.3472(4)	1	0.081(3)
Extraframework^a^						
C1	Cd	0.6667	0.3333	0.94951(19)	0.381(6)	0.0683(16)
C1A	Cd	0.6667	0.3333	0.004(3)	0.036(8)	0.11(3)
C5	Cd	1	1	1	0.123(11)	0.031(5)
C5A	Cd	1	1	0.0277(3)	0.178(7)	0.034(2)
C6	Cd	0.6667	0.3333	0.8333	0.319(6)	0.0295(14)
C8	Cd	0.5283(13)	0.4715(13)	0.0150(6)	0.049(3)	0.054(5)
CW2	Ca	0.484(7)	0.964(6)	0.484(3)	0.067(12)	0.14(4)
CW2A	Ca	0.488(3)	0.099(3)	0.4595(11)	0.056(9)	0.034(12)
CW2B	Ca	0.463(4)	0.927(4)	0.4058(18)	0.032(4)	0.03^b^
W1	O	0.602(4)	0.400(4)	0.035(2)	0.24(4)	0.12(3)
W2	O	0.609(3)	0.101(2)	0.4594(11)	0.14(2)	0.016(11)
W2B	O	0.529(4)	0.061(4)	0.4546(16)	0.23(4)	0.075(18)
W3	O	0.961(8)	0.039(8)	0.119(3)	0.17(2)	0.15^b^

^a^U isotropic

^b^fixed

**Table 5:** Atom coordinates, occupancy and atomic displacement parameters of Cd-LEV at 175 °C

Site	Scattering factor	*x*	*y*	*z*	Occ	*U*^eq^ (*Å*^2^)
T1	Si	0.6673(2)	0.0949(2)	0.89872(10)	1	0.0279(7)
T2	Si	0.75902(19)	0.00020(19)	0.00006(10)	1	0.0231(7)
T11	Si	0.5725(2)	0.9051(2)	0.10124(11)	1	0.0286(7)
O1	O	0.7025(8)	0.0232(8)	0.9438(3)	1	0.061(2)
O11	O	0.6788(7)	0.9764(8)	0.0560(3)	1	0.061(2)
O2	O	0.7600(7)	0.5203(8)	0.9004(5)	1	0.074(3)
O3	O	0.5375(6)	0.0755(7)	0.9166(3)	1	0.050(2)
O4	O	0.6671(6)	0.0484(7)	0.8330(3)	1	0.0468(18)
O5	O	0.5574(7)	0.1155(9)	0.3465(4)	1	0.074(3)
Extraframework^a^						
C1	Cd	0.6667	0.3333	0.94403(16)	0.387(7)	0.0536(16)
C1A	Cd	0.6667	0.3333	0.002(3)	0.075(7)	0.18^b^
C5	Cd	1	1	0.991(3)	0.08(2)	0.019(5)
C5A	Cd	1	1	0.9713(14)	0.17(2)	0.029(3)
C6	Cd	0.6667	0.3333	0.8333	0.306(7)	0.0276(19)
C8	Cd	0.4715(9)	0.9428(9)	0.9848(4)	0.075(3)	0.039(4)
CW2	Ca	0.716(5)	0.852(5)	0.870(2)	0.118(10)	0.15^b^
CW2A	Ca	0.765(4)	0.939(5)	0.874(2)	0.028(8)	0.01(2)
W2	O	0.587(11)	0.792(11)	0.928(5)	0.053(15)	0.04^b^

^a^U isotropic

^b^fixed

Between 200 and 250 °C, in correspondence of the pronounced drop of the unit-cell volume (Fig. 1a), the interpretation of the diffraction data was complicated by the strong smearing and broadening of the reflections (Fig. S1), the intensity of which significantly decreased. Thus, a meaningful solution of these structures was not possible and only the cell parameters were determined. At 275 °C, although a high *R*_int_ (8.84%), the structure could be solved and refined in space group *R*-3. This high- temperature structure, described in the next paragraph, is assumed to be completely dehydrated.

### The dehydrated structure of Cd-exchanged levyne at 275 °C

**Fig. 3:**
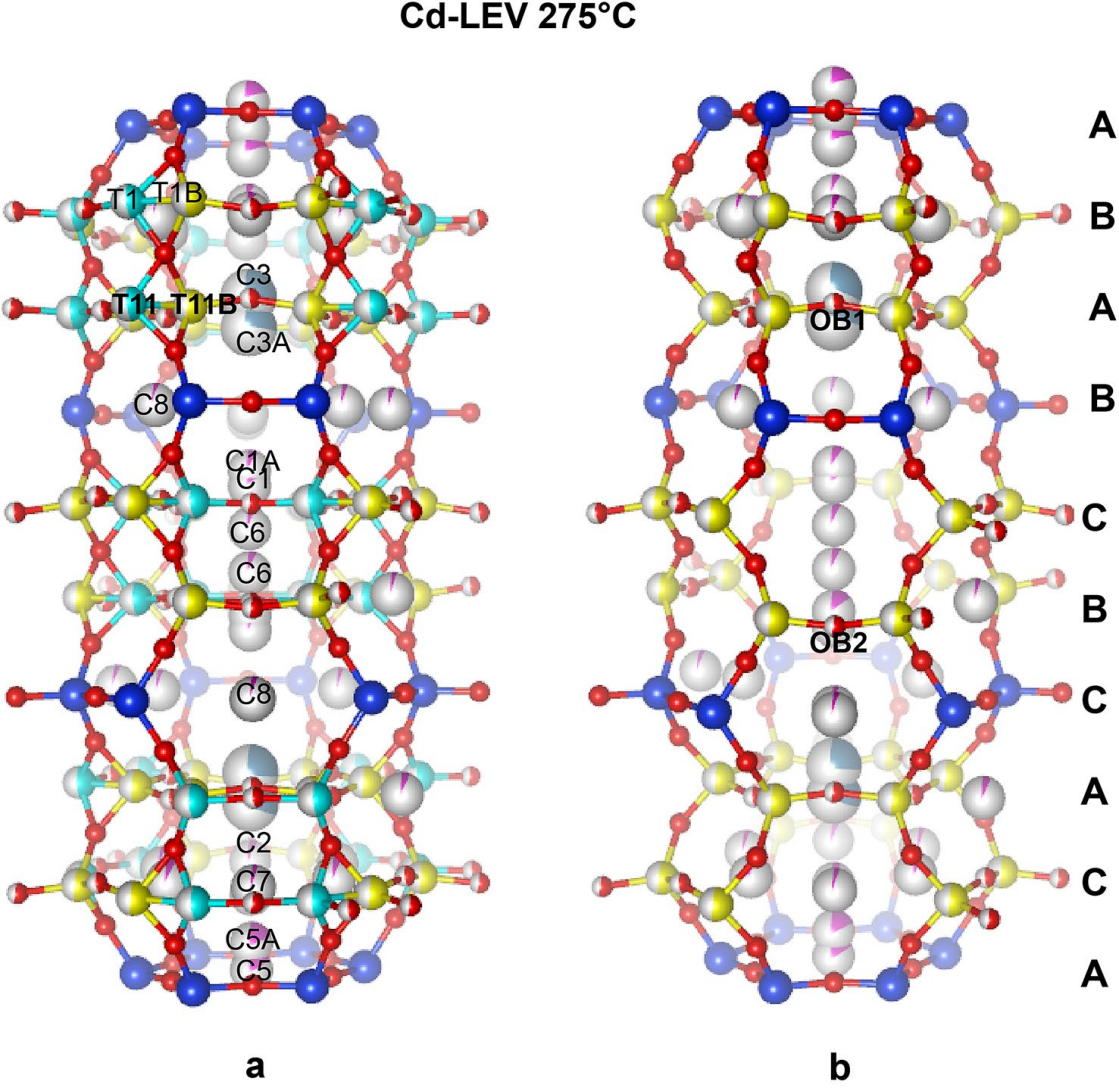
Crystal structure of Cd-exchanged levyne at 275 °C. **a** Tetrahedral sites involved in the T-O-T breaking process are reported in cyan. Yellow sites (T1B and T11B) represent the new tetrahedral sites forming as a consequence of the T–O–T rupture. **b** Structural topology (levyne B) of Cd-LEV at 275 °C. The stacking sequence of the six-membered rings along c is also shown

The contracted phase of Cd-LEV has a structural topology similar to that of levyne-Ca between 200 and 250 °C. This structure is characterized by the statistical rupture of the T–O–T connections (T1–O2–T11 and T1–O3–T11) of the D6R tetrahedra (Fig. 3a, Table 6). This mechanism involves the partial migration of T1 and T11 sites toward new position (T1B and T11B), with corresponding formation of two new tetrahedra with oxygen apices OB1 and OB2. Since this is a statistical process, two topologies coexist at high temperature: the original levyne (LEV) and the levyne B (Arletti et al.^2013^). The latter, which originates if only the new tetrahedral sites (i.e. T1B and T11B) and T2 are considered, has ABABCBCAC stacking sequence (Fig. 3b). According to the structural refinement of Cd-LEV, 50% of the original tetrahedral sites (T1 and T11) migrated to the new positions T1B and T11B. The EF cations spread mainly along the threefold axis and residual Cd is found close to the wall of the lev cavity at C2, C7 and C8 sites (Table 6). Since it was not possible to monitor the structural changes between 200 and 250 °C, it remains enigmatic whether the rupture, similar to levyne-Ca, already began at lower temperatures and gradually increased up to 275 °C, or it was a more abrupt change. A plausible hypothesis is that the strong smearing and broadening of the reflections along the c-axis (Fig. S1) observed at 200 °C, might be associated with the onset of the breaking process.

**Table 6:** Atom coordinates, occupancy and atomic displacement parameters of Cd-LEV at 275 °C

Site	Scattering factor	*x*	*y*	*z*	Occ	*U*^iso^(*Å*^2^)
T1	Si	0.6696(6)	0.0843(6)	0.8902(3)	0.465(4)^a^	0.0718(16)^b^
T1B	Si	0.5862(6)	0.9184(6)	0.8887(2)	0.535(4)^a^	0.0718(16)^b^
T2	Si	0.7489(3)	1.0000(3)	0.00005(11)	1	0.0579(14)
T11	Si	0.5851(6)	0.9160(6)	0.1097(3)	0.465(4)^a^	0.0718(16)^b^
T11B	Si	0.6675(6)	0.0814(6)	0.1113(2)	0.535(4)^a^	0.0718(16)^b^
O4	O	0.6662(8)	0.0095(10)	0.8335(3)	1	0.112(4)
O1	O	0.6793(9)	0.9955(10)	0.9425(3)	1	0.130(4)
O3	O	0.5390(17)	0.0800(18)	0.8923(8)	0.465(4)^a^	0.102(6)
O2	O	0.7707(17)	0.5413(17)	0.8875(8)	0.535(4)^a^	0.126(7)
O11	O	0.6835(10)	0.0045(10)	0.0577(4)	1	0.131(4)
O5	O	0.5515(9)	0.1034(9)	0.3320(5)	1	0.128(4)
OB1	O	0.7807(18)	0.2180(18)	0.1223(8)	0.465(4)^a^	0.109(7)
OB2	O	0.4514(17)	0.9027(18)	0.8958(8)	0.535(4)^a^	0.125(6)
Extra framework						
C1	Cd	0.6667	0.3333	0.911(2)	0.15(2)	0.070(6)
C1A	Cd	0.6667	0.3333	0.929(3)	0.07(3)	0.046(12)
C2	Cd	0.8805(15)	0.1207(14)	0.1159(7)	0.073(4)	0.108(8)
C3	Ca	1	1	0.1988(5)	0.324(17)	0.048(5)
C3A	Ca	1	1	0.2362(9)	0.30(2)	0.082(8)
C4	Cd	1	1	0.0889(18)	0.049(4)	0.11^c^
C5	Cd	1	1	0	0.175(15)	0.091(8)
C5A	Cd	1	1	0.0318(4)	0.184(8)	0.054(4)
C6	Cd	0.6667	0.3333	0.8056(16)	0.065(8)	0.112(17)
C7	Cd	0.869(2)	0.736(3)	0.8846(11)	0.029(3)	0.064(11)
C8	Cd	0.5326(17)	0.0655(17)	0.0023(8)	0.047(3)	0.080(7)

^a^Occ. T1 = Occ. T11 = 1−Occ.T1B = 1−Occ.T11B = Occ.OB1 = 1−Occ.OB2 = Occ. O3 = 1−Occ.O2

^b^Constrained to be equal

^c^Fixed

To check if this structural configuration is maintained at higher temperature, tentative refinements were performed on the structures at 350 and 375 °C. Although the low quality of the data, characterized by low resolution, no additional changes with respect the structure at 275 °C were noticed. As an example, the refined parameters of the structure at 350 °C are reported in Table S2a, b.

**Fig. 4:**
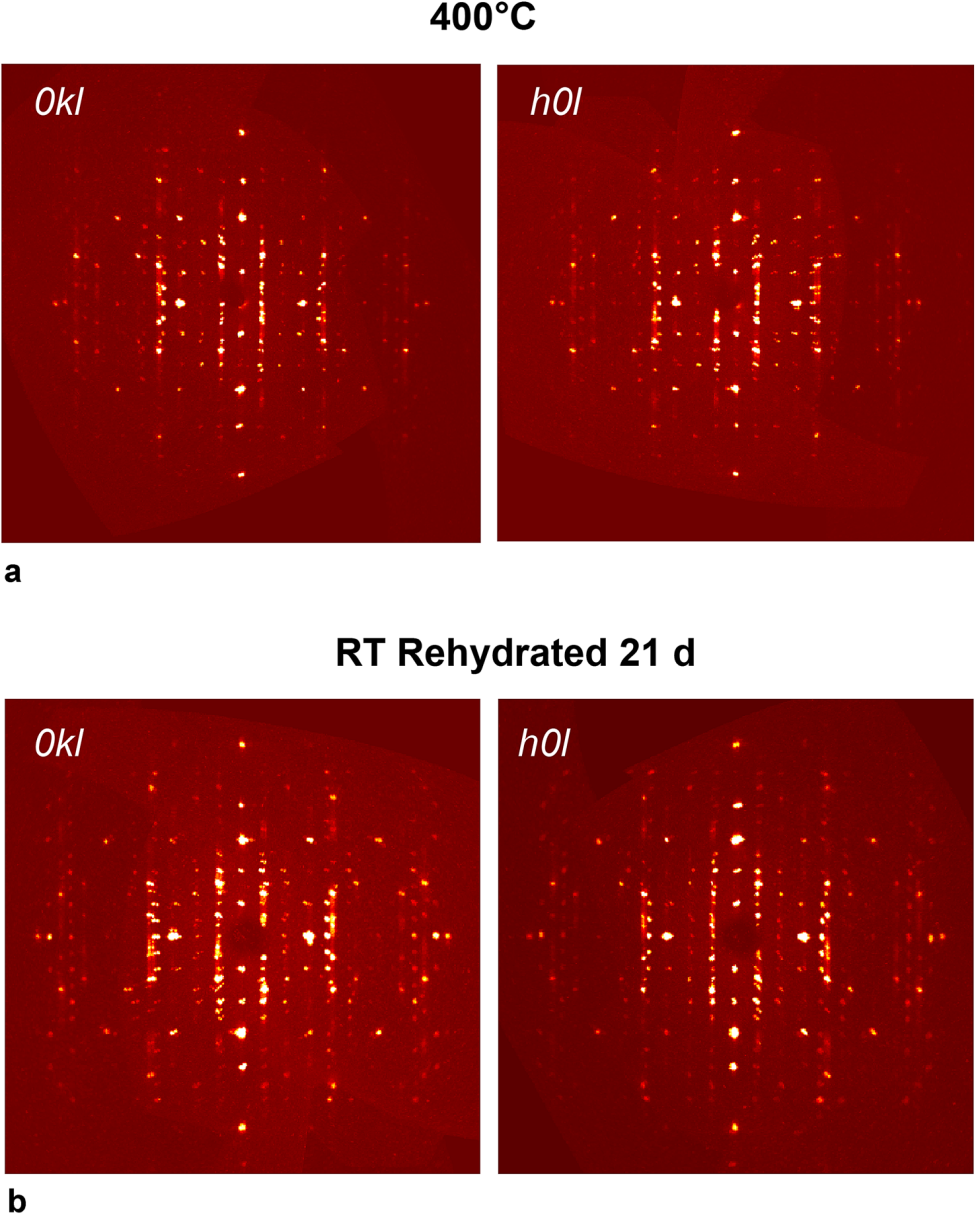
Reconstructed precession images of 0kl* and h0l* layers (c*-axis is vertical) of Cd-LEV collected at 400 °C and at RT after rehydration

Diffraction data at 400 °C (Fig. 4a) consisted of very broad reflections with a maximum resolution of 1.0 Å, pointing out the onset of the structural collapse. The estimated unit-cell volume determined at this temperature was 3226(1) Å^3^. The determination of the correct unit-cell parameters was complicated not only because of the additional damping of the atomic scattering-factors at high temperature but also because of the strong smearing and splitting of the reflections (Fig. 4a). Such a contracted structure, as expected from the extent of the structural changes, is not able to reabsorb water. Diffraction data collected on the crystal used for the HT measurements and subsequently exposed to high humidity conditions at room temperature were limited to 2*θ* = 41.7° and are characterized by smeared and weak reflections (Fig. 4b). The unit-cell volume (3198.8(9) Å^3^) was comparable with that obtained for the structure at 400 °C, confirming that it could not re-hydrate.

### Differences with levyne- Ca and Ag-exchanged levyne

The structural evolution of Cd-LEV upon continuous heating can be summarized by the following two steps: (i) initial dehydration with corresponding diffusion of the EF cations within the zeolitic cavities; (ii) pronounced contraction of the unit-cell volume associated with the rupture of 50% of the T–O–T bonds constituting the D6R cage. This step leads the so-called levyne B topology (Arletti et al. 2013). Thus, differently from the pristine material levyne-Ca (Cametti, 2018), no additional transition to the levyne B’phase occurs in Cd-exchanged levyne.

In levyne-Ca, this transformation is associated with the breaking of an additional T–O–T bridge of the framework (T2–O5–T2), which starts at 275 °C in correspondence of the loss of residual H_2_O. It may be argued that, in levyne-Cd, the onset of the breaking of an additional T–O–T was not detectable due to the quality of the diffraction data (Table 1). However, two arguments support a different transformation path:

- in levyne-Ca, at 300 °C, the migration of (Si,Al) atoms toward the new T1B site amounted to 37%, differently from Cd-LEV where at 275 °C the population of the new T sites (T1B and T11B) is 50%;
- the pronounced difference (from 200 °C on) between the unit-cell volume curves (Fig. 1a) indicates that in Cd-LEV the dehydration process is not only shifted toward lower temperatures (e.g. the unit-cell volume of the structure at 225 °C corresponds to that of levyne-Ca at 275 °C), but also more drastic in terms of structural contraction. Thus, it is plausible that such behaviour is associated with different phase transformations.

It has to be pointed out, at this point, that the drastic contraction of the unit cell of Cd-LEV is also related to a reduced thermal stability. Because of the smaller size (and higher ionic potential) of Cd^2+^ with respect to both Ca^2+^ and Ag^+^, it is reasonable that the thermal stability of Cd-LEV is lower compared to Ca- and Ag-LEV (Cruciani 2006). In this sense, Cd^2+^ exerts more strain on the framework that causes an earlier collapse with respect to Ca-LEV.

Interestingly, similar to Cd-LEV, upon dehydration levyne B’ structure does not form in Ag-LEV too (Cametti et al. 2020). In that case, the absence of the B’ topology was explained based on the H_2_O content of the RT structure. Ag-LEV at ambient conditions contains less H_2_O compared to the pristine material; therefore, a second dehydration step, which would trigger the B-B’ transition, does not take place. In Cd-LEV, the number of H_2_O pfu determined by the structural refinement (Table 1) is even slightly higher than the corresponding one in levyne-Ca. This is not surprising, considering that, in natural levyne-Ca, Na^+^ and K^+^ are also present as EF cations. In Cd-LEV, only divalent cations occupy the zeolitic cavities, where, consequently more H_2_O can be accommodated. Nevertheless, the structure does not experience a second T–O–T rupture. Thus, the missing transformation to B’ must be related to specific properties of Cd^2+^.

In the SR-XRPD study by Arletti et al. (2013), this additional modification was not reported as well. In this case, the kinetic of the experiments (faster and far from the equilibrium) was responsible for the rapid water loss and the structure had not enough time to complete the transformations. In our SC-XRD experiments, it seems like a similar mechanism is driving the dehydration of Cd-LEV. That is, a faster (compared to levyne-Ca) release of water may lead to a more abrupt dehydration and, therefore, to the absence of the B- > B’ transformation. The main difference between the trend of the unit-cell volume of levyne-Ca and Cd-LEV in Fig. 1a is certainly the stronger contraction of Cd-LEV. However, Cd-LEV is also characterized by a steeper unit-cell volume curve, pointing to a sudden water loss similar to that observed by Arletti et al. (2013). Hence, Cd^2+^ may play a double role in such process as follows: (i) it tends to coordinate less H_2_O than Ca^2+^ due to the smaller radius; therefore, the H_2_O are released earlier at lower temperature; (ii) it exerts more strain on the framework, which starts collapsing already at 400 °C.

## Conclusions

A variety of factors influences the response of natural zeolites to the heating stimuli, complicating the prediction of their behaviour upon dehydration. Because of the wide range of applications where these minerals are used, knowing the structural transformations occurring as a function of increasing temperature is of great interest. In this study, the investigation of a partially exchanged Cd-levyne demonstrated that, although residual Ca ions are present in the structure, the dehydration behaviour significantly differs from that of the natural material, levyne-Ca. From 50 to 175 °C, a similar water release and associated diffusion path of the EF cations is observed for Ca- and Cd-LEV. However, from 200 °C Cd-LEV follows a different dehydration path. The most relevant outcome is the lack of the structural transformation to the levyne B’ topology, as detected in levyne-Ca, and, most important, the reduced thermal stability of Cd-LEV, which starts collapsing already at 400 °C. This is undoubtedly related to the presence of Cd^2+^ and to the strain exerted on the framework. It is worth mentioning that a similar behaviour, i.e. reduced thermal stability associated with a pronounced contraction of the unit-cell volume upon heating, was also reported for another zeolite, stellerite, previously exchanged with Cd^2+^ (Cametti et al. 2019).

## Supplementary Information

Below is the link to the electronic supplementary material.

Supplementary file1 (DOCX 661 KB)

Supplementary file2 (CIF 331 KB)

Supplementary file3 (CIF 330 KB)

Supplementary file4 (CIF 332 KB)

Supplementary file5 (CIF 327 KB)

Supplementary file6 (CIF 299 KB)

Supplementary file7 (CIF 290 KB)

## Data Availability

All data generated or analysed during this study are included in this published article (and its supplementary information files).
