# Supplementary material for: Stepwise dehydration of Cd-exchanged levyne: thermal stability and structural modifications
Source: Phys Chem Miner. 2021 May 12;48(6):23. doi: 10.1007/s00269-021-01146-6 (PMC8550444; doi:10.1007/s00269-021-01146-6)
Supplement: Supplementary file 1 — Supplementary file1 (DOCX 661 KB) [file 269_2021_1146_MOESM1_ESM.docx]

**Stepwise dehydration of Cd-exchanged levyne: thermal stability and structural modifications**

Georgia Cametti^1^

^1^ Institute of Geological Sciences, Baltzerstrasse 1+3, 3012 Bern, Switzerland

**Supporting Information**

**Tables**

**Table S1** Atom coordinates occupancy and atomic displacement parameters of Cd-LEV at RT

| *Site* | *Scattering factor* | *x* | *y* | *z* | *Occ.* | *U^eq^(Å^2^)* |
| --- | --- | --- | --- | --- | --- | --- |
| T1 | Si | 0.43813(13) | 0.10466(13) | 0.40464(7) | 1 | 0.0220(5) |
| T2 | Si | 0.23671(15) | 0 | 0.5 | 1 | 0.0204(6) |
| O1 | O | 0.3174(4) | 0.0250(4) | 0.4409(2) | 1 | 0.0361(11) |
| O2 | O | 0.4925(6) | 0.2462(3) | 0.4191(3) | 1 | 0.0320(14) |
| O3 | O | 0.5366(3) | 0.0731(5) | 0.4272(3) | 1 | 0.0297(14) |
| O4 | O | 0.4106(6) | 0.0773(6) | 0.3333 | 1 | 0.0379(15) |
| O5 | O | 0.1096(3) | -0.1096(3) | 0.4839(3) | 1 | 0.0370(16) |
| C1 | Cd | 0.6667 | 0.3333 | 0.4716(8) | 0.32(8) | 0.021(3)* |
| C1A | Cd | 0.6667 | 0.3333 | 0.4789(7) | 0.48(8) | 0.037(2)* |
| C3 | Ca | 0 | 0 | 0.4009(19) | 0.30(2) | 0.15^‡^ |
| C4 | Ca | 0 | 0 | 0.283(3) | 0.18(2) | 0.15^‡^ |
| C5 | Cd | 0 | 0 | 0.5 | 0.216(8) | 0.026(2)* |
| W2 | O | 0.0889(9) | -0.1484(9) | 0.3760(4) | 0.384(17) | 0.028(4)* |
| W4 | O | 0.2115(14) | -0.2115(14) | 0.3794(13) | 0.88(6) | 0.212(19)* |
| W1 | O | 0.8160(7) | 0.4080(4) | 0.5436(4) | 1 | 0.065(3) |
| W3 | O | -0.0765(17) | 0.0765(17) | 0.3480(16) | 0.93(7) | 0.26(2)* |
| *U isotropic  ^‡^ fixed | | |  |  |  |  |

**Table S2a** Crystal data and refinement parameters of Cd-LEV 350°C

| **Crystal data** | **Cd-LEV 350C** |
| --- | --- |
| *a* (Å) | 12.6031(10) |
| *c* (Å) | 23.5420(19) |
| *V* (Å^3^) | 3238.4(6) |
| *Z* | 3 |
| Space Group | *R-*3*m* |
| Refined Chemical formula | Cd_2.11_Ca_1.4_(Si, Al)_18_O_36_ |
| Crystal size (mm) | 0.10 × 0.12 × 0.13 |
| **Intensity measurement** |  |
| Diffractometer | BRUKER APEX II SMART |
| X-ray radiation | MoKα λ = 0.71073 Å |
| X-ray power | 50 kV, 30 mA |
| Monochromator | graphite |
| Temperature (°C) | 350 |
| Exposure time (s) | 10 |
| Max. 2θ (°) | 43.51 |
| Index ranges | -13 ≤ *h* ≤ 13 |
|  | -13 ≤ *k* ≤ 13 |
|  | -24 ≤ *l* ≤ 23 |
| No. of measured reflections | 7274 |
| No. of unique reflections | 860 |
| No. of observed reflections I > 2σ (I) | 511 |
| **Structure refinement** |  |
| No. of parameters used in the refinement | 90 |
| *R*(int) | 0.0786 |
| *R*(σ) | 0.0441 |
| GooF | 1.579 |
| *R*1, *I*>2σ (*I*) | 0.1529 |
| *R*1, all data | 0.1972 |
| *wR*2 (on *F*^2^) | 0.4282 |
| Δρ_max_ (eÅ^-3^) close to | 0.85 C8 |
| Δρ_min_ (-eÅ^-3^) close to | -1.19 C3 |

**Table S2b** Atom coordinates, occupancy and atomic displacement parameters of Cd-LEV at 350°C

| *Site* | *Scattering factor* | *x* | *y* | *z* | *Occ.* | *U^iso^(Å^2^)* |
| --- | --- | --- | --- | --- | --- | --- |
| T1 | Si | 0.6698(7) | 0.0835(7) | 0.8896(3) | 0.454(4)* | 0.072(2) ^‡^ |
| T1B | Si | 0.5875(6) | 0.9199(6) | 0.8886(3) | 0.546(4)* | 0.072(2) ^‡^ |
| T2 | Si | 0.7484(3) | 0.9999(3) | 0.00005(12) | 1 | 0.0582(18) |
| T11 | Si | 0.5860(7) | 0.9166(7) | 0.1103(3) | 0.454(4)* | 0.072(2) ^‡^ |
| T11B | Si | 0.6679(6) | 0.0802(6) | 0.1113(3) | 0.546(4)* | 0.072(2) ^‡^ |
| O4 | O | 0.6669(9) | 0.0058(11) | 0.8334(4) | 1 | 0.117(4) |
| O1 | O | 0.6785(10) | 0.9982(11) | 0.9436(4) | 1 | 0.122(4) |
| O3 | O | 0.539(2) | 0.077(2) | 0.8928(9) | 0.454(4)* | 0.113(7) |
| O2 | O | 0.7697(17) | 0.5394(19) | 0.8903(9) | 0.546(4)* | 0.123(7) |
| O11 | O | 0.6809(10) | 0.0022(11) | 0.0566(4) | 1 | 0.124(4) |
| O5 | O | 0.5514(10) | 0.1019(10) | 0.3334(5) | 1 | 0.128(4) |
| OB1 | O | 0.781(2) | 0.218(2) | 0.1236(9) | 0.454(4)* | 0.106(7) |
| OB2 | O | 0.4524(18) | 0.9051(19) | 0.8961(9) | 0.546(4)* | 0.122(7) |
| **Extraframework** | | | | | | |
| C1 | Cd | 0.6667 | 0.3333 | 0.908(3) | 0.14(3) | 0.078(9) |
| C1A | Cd | 0.6667 | 0.3333 | 0.928(4) | 0.08(3) | 0.054(15) |
| C2 | Cd | 0.8780(15) | 0.1219(15) | 0.1139(7) | 0.071(4) | 0.099(8) |
| C3 | Ca | 1 | 1 | 0.1998(6) | 0.37(2) | 0.053(6) |
| C3A | Ca | 1 | 1 | 0.2356(10) | 0.33(2) | 0.080(9) |
| C4 | Cd | 1 | 1 | 0.093(2) | 0.041(4) | 0.11^§^ |
| C5 | Cd | 1 | 1 | 0 | 0.181(18) | 0.081(9) |
| C5A | Cd | 1 | 1 | 0.0311(5) | 0.191(11) | 0.061(5) |
| C6 | Cd | 0.6667 | 0.3333 | 0.797(3) | 0.074(14) | 0.14(2) |
| C7 | Cd | 0.870(4) | 0.738(4) | 0.8799(16) | 0.027(3) | 0.083(18) |
| C8 | Cd | 0.5341(18) | 0.0673(17) | 0.0027(8) | 0.048(3) | 0.073(8) |
| *Occ. T1 = Occ. T11 = 1-Occ. T1B =1-Occ. T11B = Occ. OB1= Occ. 1-OB2 = Occ. O3 = Occ. 1-O2  ^‡^ constrained to be equal  ^§^ fixed | | | | | | |

**Figures**

**Fig. S1** Reconstructed precession images of levyne-Cd at 200 and 250°C. The c*-axis is vertical

**
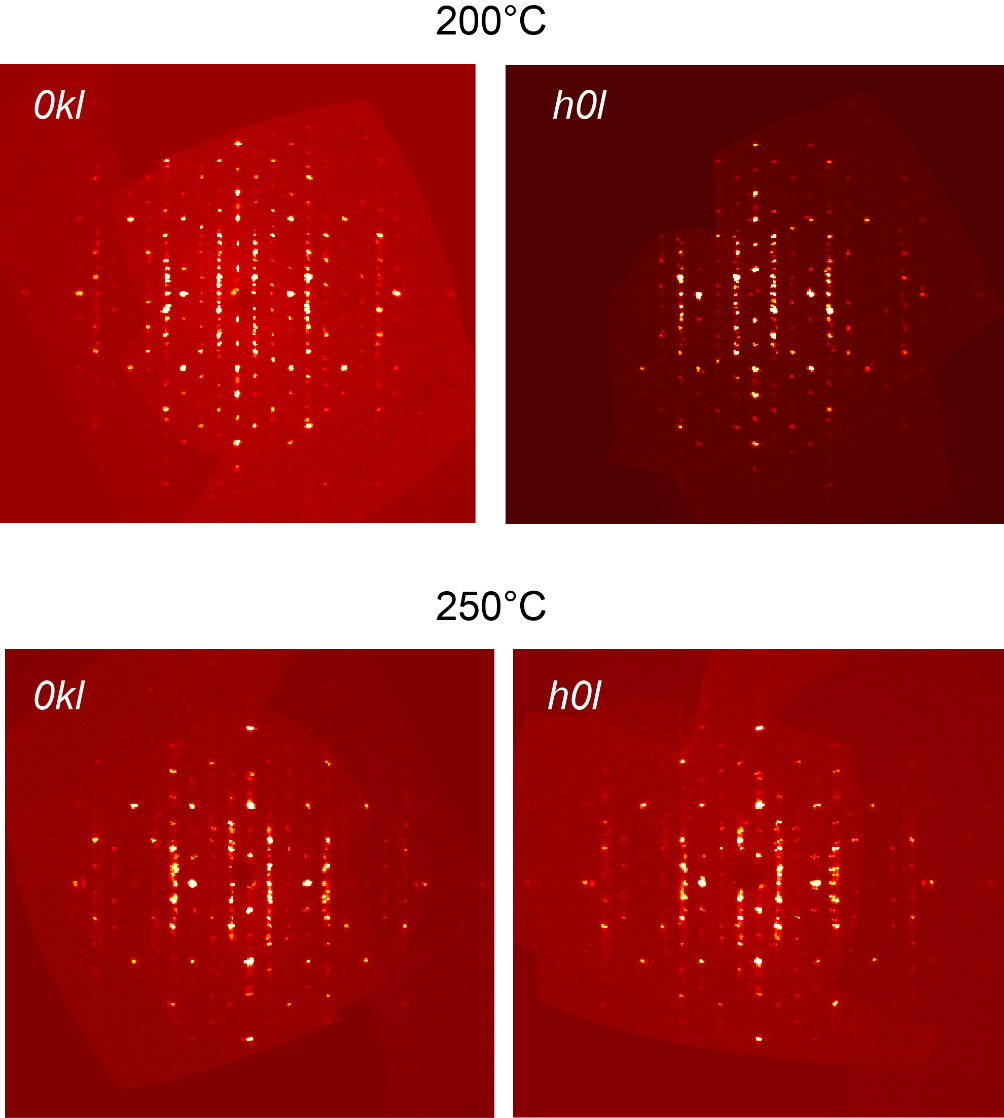
**
